# Er:YAG Laser Versus Sandblasting for Build-Up Conditioning in Adhesive Cementation: A Retrospective Study of 187 Posterior Indirect Restorations

**DOI:** 10.3390/dj14010034

**Published:** 2026-01-05

**Authors:** Ilaria Giovannacci, Giuseppe Pedrazzi, Beatrice Spaggiari, Paolo Vescovi

**Affiliations:** 1 Oral Medicine and Oral Laser Surgery Unit, Department of Medicine and Surgery, University of Parma, Via Gramsci 14, 43125 Parma, Italy; beatricespaggiari@gmail.com (B.S.); paolo.vescovi@unipr.it (P.V.); 2 Private Practice, COeS (Centro Odontoiatrico e Stomatologico), Borgo Cantelli, 11, 43121 Parma, Italy; 3 UniCamillus-Saint Camillus International University of Health Sciences, 00184 Rome, Italy; 4 Unit of Neuroscience, Department of Medicine and Surgery, Plesso Biotecnologico Integrato, University of Parma, 43125 Parma, Italy; giuseppe.pedrazzi@unipr.it; 5 Interdepartmental Center of Robust Statistics (Ro.S.A.), University of Parma, 43125 Parma, Italy

**Keywords:** adhesive indirect restoration, sandblasting, Er:YAG laser, adhesive cementation, build-up

## Abstract

**Background**: Adhesive indirect restorations have become increasingly common in daily clinical routine in most dental practices. Before etching and adhesive application, a sandblasting procedure is essential to clean and increase the microporosity of the surface. Air abrasion with aluminum oxide particles significantly improves the bond strength. However, this procedure may have some limitations, such as the presence of powder particles. Recently, the Er:YAG laser in QSP mode has been proposed for conditioning build-ups prior to adhesive cementation. The aim of this study was a retrospective analysis of adhesive indirect restoration in which build-up was conditioned or using a traditional sandblaster with alumina powder or using the Er:YAG laser in QSP mode. **Methods**: 187 posterior indirect adhesive restorations were cemented using two different conditioning techniques: in 96 cases (51.34%) build-up conditioning was performed using an intraoral sandblaster with alumina oxide (Microetcher CD, Kavo, Biberach, Germany); in 91 cases (48.66%) build-up conditioning was performed using the Er:YAG laser (Fotona LighWalker^®^, Ljubljana, Slovenia) in QSP modality (1 W, 10 Hz, 100 mJ). The clinical efficacy of the two techniques was evaluated and compared, assessing the occurrence of complications such as debonding, fracture, secondary leakage, and hypersensitivity over time. **Results**: The frequency of secondary complications was very low in both groups. Only one case of debonding and one case of restoration cracking was observed in the sandblasting group, with none in the laser group (*p* = 0.329). Secondary caries occurred in both groups. A difference was observed in postoperative hypersensitivity: 6% in the sandblasting group and 1% in the laser group (*p* = 0.064). The Kaplan–Meier curves of the two conditioning techniques showed comparable survival over time (Log-rank test χ^2^ = 2.4864/*p* = 0.1148). The mean follow-up was 30 months. **Conclusions**: The success rates of these restorations are very high if adhesive cementation steps are properly followed. Conditioning the build-up before etching is essential. Among these, the Er:YAG laser in QSP mode seems to provide excellent results in the absence of dust and smear layer. Recurrence rates of complications such as decementation, leakage, and cracking resulted in less than 1%. Furthermore, it is interesting to note that using the laser to condition the build-up appears to reduce the recurrence of post-cementation hypersensitivity. These data require confirmation through prospective clinical trials.

## 1. Introduction

In recent years, adhesive techniques and CAD/CAM materials have evolved significantly. This allows us to perform indirect adhesive restorations extremely effectively but at the same time are minimally invasive. For these reasons, adhesive indirect restorations have become increasingly common in daily clinical routine in most dental practices. Overlays are particularly indicated where partial or total cusp coverage is required [1,2].

The step-by-step procedure first requires the opening of the cavities, the removal of any previous restorations and carious lesions, the evaluation of enamel and dentin thickness and the consequent reduction in unsupported tissue [3].

Then, composite build-up with immediate dentin sealing (IDS) and, if necessary, cervical margin repositioning (CMR) is performed. At this point, build-up is prepared according to new modified principles (morphology-guided preparation technique—MDPT). A definitive impression must be taken for the realization of the definitive manufacture (composite or ceramic). Fitting, contact points, occlusion and aesthetic integration of the restoration are checked before the isolation with the rubber dam and the adhesive cementation [3].

The success of these restorations depends on various factors, including the quality of the preparation, the type of luting agents used, the cementation technique and the thickness of the final restoration [4,5,6,7].

Regarding adhesive cementation process, it is known in the literature that it must be performed properly to allow correct adhesion of the overlay and avoid complications such as secondary infiltration, cracking or decementation. The steps for adhesive cementation consist of steps performed inside the overlay and steps on the build-up. The steps on the surface of the overlay depend on the material used; the steps on the build-up are always the same and performed under rubber dam isolation. Before etching with 37% orthophosphoric acid and application of the adhesive system, a sandblasting procedure is essential to clean the build-up and increase the microporosity of the surface.

The most widely used method in the literature is air abrasion with aluminum oxide particles propelled by compressed air at high speed [8], which, once they reach the prepared surface, cause a morphological modification [9]. This technique creates microporosity on the surface, increasing the surface area and improving the wettability of adhesive systems. Air abrasion is influenced by factors such as particle size, air pressure, the distance between the tip of the instrument and the surface, the angle of incidence, and the duration of application [8]. Do Nascimento Santos et al. in 2025 performed a systematic review and meta-analysis in order to investigate the effect of aluminum oxide air abrasion on the bond strength of resin cements to indirect restorations [2]. The authors concluded that air abrasion with aluminum oxide particles (50 µm) is an effective method, significantly improving the bond strength of resin to indirect restorations compared to no use and pumice paste [2].

However, this procedure may have some limitations. When air abrasion was applied to immediate dentin sealant (IDS) substrates, the bond strength was reduced compared to the control [2,6,10].

Furthermore, the presence of micro powders may be unsafe for the patient, uncomfortable for the operator, and could interfere with the bonding process.

Among new technologies, there are lasers that have an affinity for hard tissue and that are used on dental tissue. Among these, the Er:YAG (Erbium-doped: Yttrium–aluminum–garnet) laser is widely used both for cavity preparation and for enamel and dentin conditioning in adhesive procedures due to its wavelength of 2940 nm absorbed by water and hydroxyapatite.

For some years, Quantum Square Pulse (QSP) mode has been added as one of the Er:YAG laser pulse modes. A standard laser pulse is divided into five super short “pulses” (quanta pulses). The time between pulses is 85 microseconds (pulset spacing) and the pulse duration is 50 μsec. It means that the consistent debris cloud has not yet formed, reducing scattering to a minimum [11].

Lukac et al. analyzed enamel and dentin surfaces after preparation with QSP and appear high-quality for high bond strength and are free of smear layers. Dentin appears clean, regular, and flat with well-opened tubules, without differences between intertubular and peritubular dentin. Enamel appears clean and uniform with well-defined microroughness [12].

In a recent ex vivo study, the authors analyzed the effectiveness of the Er:YAG laser in QSP mode for conditioning build-ups prior to adhesive cementation [13]. In this study, the build-up treated with the laser were compared with surfaces treated using a traditional sandblaster (alumina oxide powder) and with a control group (untreated surfaces, only prepared with burs). Surface roughness was analyzed with a profilometer, and surface morphology was evaluated under an Environmental Scanning Electron Microscopy (ESEM). The results showed that the roughness of the laser group was significantly greater than that of the sandblaster group and control group, both in dentin, enamel, and resin [13].

The laser parameters tested in the ex vivo study with water spray were chosen based on a careful analysis of the literature. Among the effectives ones, the lowest power and energy values (1 W; 100 mJ; 10 Hz) were chosen. Furthermore, when applied in QSP mode, they maintain the same efficacy, but the energy is fragmented into five micropulses (20 mJ repeated five times). This results in the same effect, but reduced scattering and thermal effects, making the technique extremely effective but safer than any other setting [14].

ESEM analysis revealed a large quantity of smear layer in the sandblaster and control groups. In the laser group, there was no debris, tubules were present in the dentin, and well-organized prisms with a honeycomb appearance were present in the enamel, almost simulating an etching action. No damage was detected in the surfaces treated with the QSP laser, making them particularly favorable for adhesion [13].

The results of the ex vivo study were extremely encouraging and useful to further validate the parameters used. The aim of this study was to investigate in vivo the efficacy of this application by retrospective analysis of 187 adhesive indirect restoration in which build-up was conditioned or using a traditional sandblaster with alumina powder or using the Er:YAG laser in QSP mode.

The clinical efficacy of the two techniques was evaluated and compared, assessing the occurrence of complications such as debonding, fracture, secondary leakage, and hypersensitivity over time.

## 2. Materials and Methods

This retrospective study analyzed 187 posterior indirect adhesive restorations cemented using two different build-up conditioning techniques.

The treatment was carried out in the accordance with the principles of the Declaration of Helsinki.

**Figure 1:**
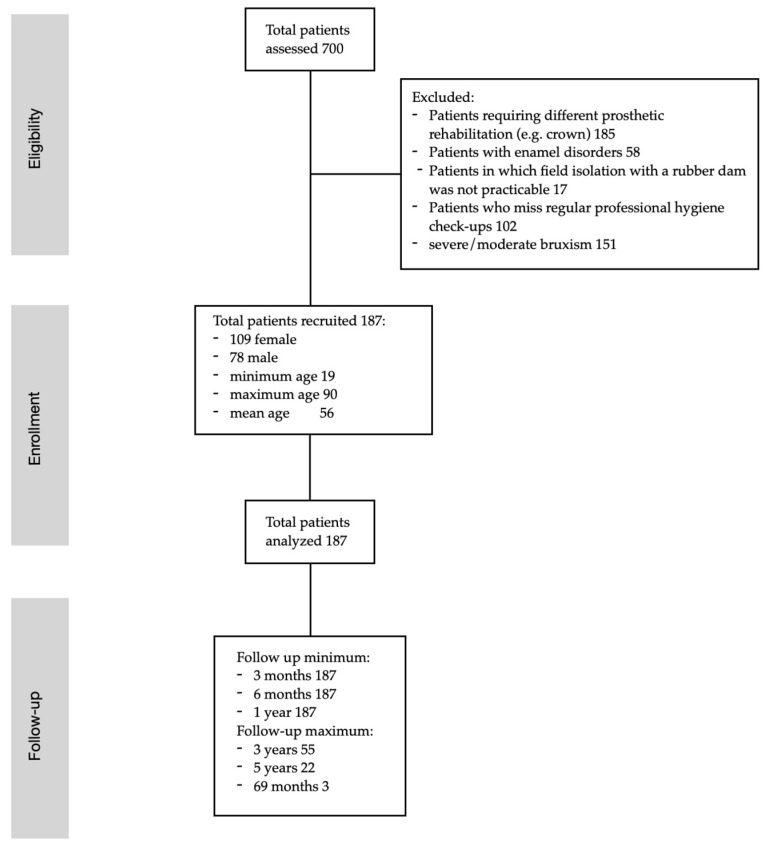
Strobe flow diagram summarizing inclusion/exclusion criteria of patients in the study.

All adult patients capable of giving consent to treatment were considered for the study. Patients requiring indirect restorations of the posterior sectors (molars/premolars) of both the upper and lower jaws were enrolled. Patients with enamel anomalies and patients who did not have regular professional hygiene sessions were excluded from the study. Similarly, patients with severe/moderate bruxism and teeth that could not guarantee adequate isolation with a rubber dam were not enrolled (Strobe Flow Diagram, Figure 1).

The primary outcome of the study was to evaluate the recurrence of complications following cementation in order to investigate whether clinical efficacy differs on the basis of the build-up sandblasting technique used.

FDI criteria are currently the accepted global standard for evaluating dental restorations (Table 1). Out of total FDI properties, 5 properties were selected due their relevance to this study [15].

The properties selected were decementation, recurrence of caries (secondary decay), postoperative sensitivity, tooth vitality (need for root remote root canal therapy) and tooth integrity (crack).

All patients were assessed at baseline, three months, six months and one year (followed by annual follow-ups). At baseline and three months, sensitivity was tested using an air stimulus and the results were assessed using the Schiff scale.

The scale was scored as follows:

1. Subject does not respond to air stimulus
2. Subject responds to air stimulus but does not request discontinuation of stimulus
3. Subject responds to air stimulus and requests discontinuation or moves from stimulus
4. Subject responds to air stimulus, considers stimulus to be painful, and requests discontinuation of the stimulus [16].

**Figure 2:**
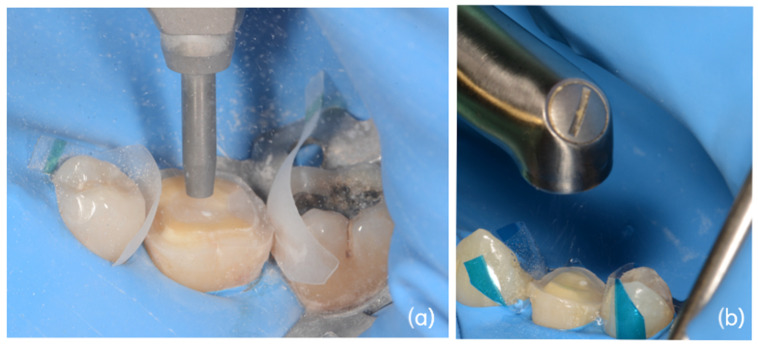
Build-up surface treatment. (**a**) Sandblaster conditioning with aluminum oxide powder (30 µm particle size, 2.5 bar pressure, 10 s, 10 mm distance from tooth surface); (**b**) Er:YAG laser conditioning (QSP modality, power 1 W, frequency 10 Hz, energy 100 mJ.

In 96 cases (51.34%), build-up conditioning was performed using an intraoral sandblaster (Microetcher CD, Kavo, Biberach, Germany) at 2.5 bar pressure, approximately 10 mm from the tooth surface for 10 s. The powder used was aluminum oxide (Al_2_O_3_, mean particle size 30 μm) (Figure 2a).

In 91 cases (48.66%), build-up conditioning was performed using the Er:YAG laser (Fotona LighWalker^®^, Ljubljana, Slovenia) in QSP modality—power: 1 W, frequency: 10 Hz, energy: 100 mJ Handpiece H02 (spot 0.9 cm)—Fluence: 16.05 J/cm^2^—Power Density: 1.573 W/cm^2^ (Figure 2b).

Except for the build-up conditioning method (which is the variable this study intends to investigate), all other steps were identical in both groups, and the restorations were all performed with the same material and by the same dental technician.

**Figure 3:**
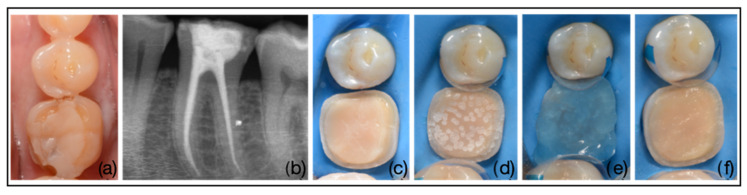
Build-up adhesion steps. (**a**) Initial clinical manifestation of carious disease with distal fracture of the clinical crown, (**b**) Endodontical treatment of the element and build-up reconstruction, (**c**) Build-up preparation according to MDPT, (**d**) Buil-up surface treatment with Er:YAG laser, (**e**) Application of 37% orthophosphoric acid on build-up surface, (**f**) Build-up surface aspect after application of two-steps adhesive system.

After conditioning the build-up (performed or with the intraoral sandblaster or with the Er:YAG laser), the cementation steps were identical in both groups. Specifically, the build-up was treated with 37% orthophosphoric acid for 15 s, then rinsed thoroughly, followed by a two-step adhesive system OptiBond FL (primer and adhesive) (Kerr Corp., Brea, CA, USA) (Figure 3).

The material of the indirect restorations was the same in all 187 cases and it is Grandio Blocs (GB, VOCO), a CAD-CAM hybrid composite resin filled with 86% ceramic nanoparticles.

Consequently, the steps performed on the internal surface of the restoration were the same in both groups. All restorations were sandblasted in the laboratory with aluminum oxide (Al_2_O_3_), 90 µm, 2 bar, for 3–4 s at a distance of 10 mm and an angle of 90°. After sandblasting, restorations were cleaned with ethanol in an ultrasonic bath for 5 min and dried with compressed air [17].

Then, 37% orthophosphoric acid and application of adhesive (OptiBond FL). The cement used in both groups was a radio-opaque light curing nano hybrid composite (Venus^®^ ART, Kulzer, Hanau, Germany). To heat the luting composite, the specific tips were placed in the special oven (Ena Heat, Composite Heating Conditioner; Micerium Group, Milan, Italy). The temperature recommended in the manufacturer’s specifications, i.e., 55 °C, was maintained for 20 min before use.

**Figure 4:**
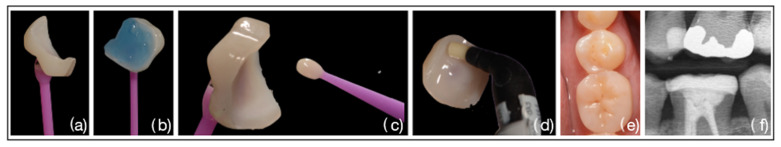
Internal surface treatment of the indirect restoration. (**a**) Laboratory sandblasting, (**b**) 37% orthophosphoric acid application, (**c**) Adhesive application, (**d**) Heated light-curing miniparticle hybrid composite application, (**e**) Final aspect of indirect restoration after cementation, (**f**) Final X-ray control.

The restorations were slowly installed by applying finger pressure until complete insertion; at this point light-curing was performed for 20 s from the occlusal, buccal, and lingual surface. Light-curing was performed using LED light-curing lamp (Valo^TM^, Ultradent, Milan, Italy) in standard mode (1000 mW/cm^2^) (Figure 4) [18].

### 2.1. Sample Description

**Figure 5:**
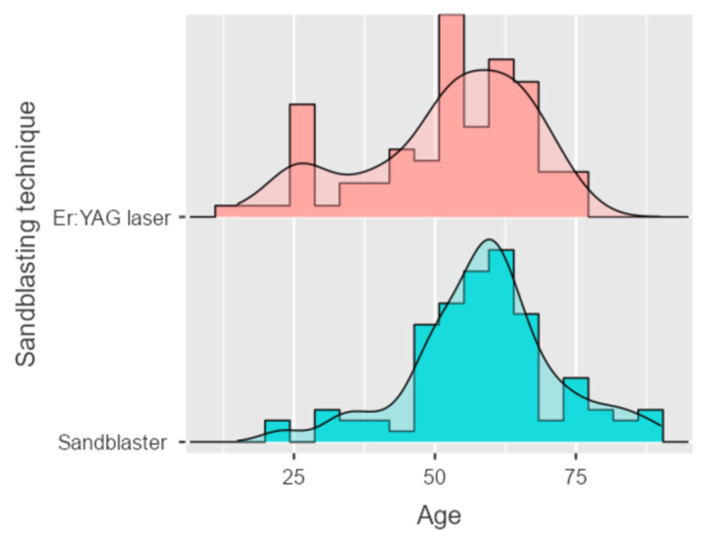
Age distribution and average age in the analyzed groups (sandblaster and Er:YAG laser).

A total of 109 out of the 187 patients (58.29%) were female while 78 (41.71%) were male, with a mean age of 56 years (minimum 19: maximum: 90). The mean age in the sandblaster group was 58; the mean age in the laser group resulted 52 (Figure 5).

Almost all restorations (*n* = 179; 95.72%) were full cusps coverage (overlay); 5 (2.67%) were inlays and 3 (1.6%) were overlay veneers.

**Figure 6:**
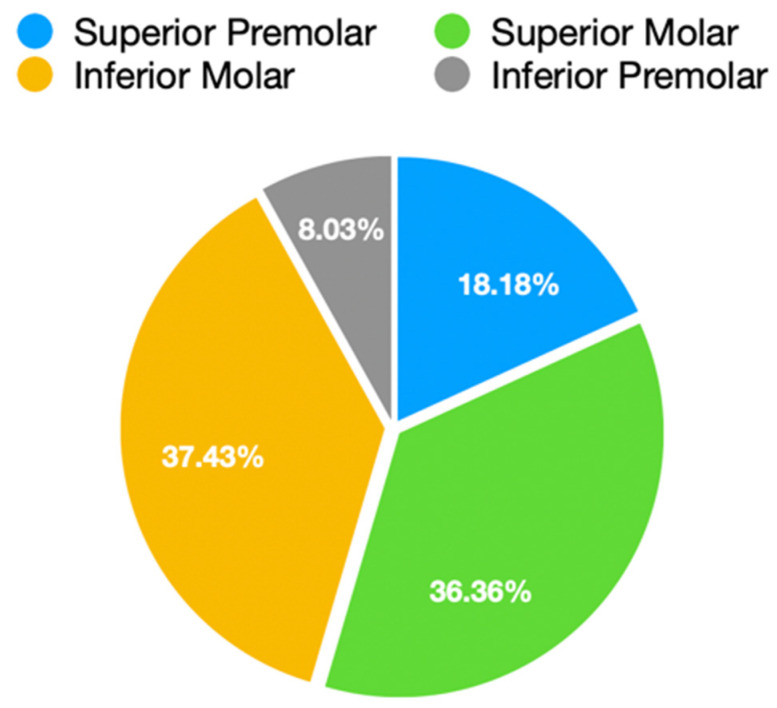
Pie chart summarizing the representation of dental elements within the sample.

Table 2 shows the frequency of the elements on which the indirect restoration was performed, and Figure 6 shows the elements grouped by sector. In particular, the most represented are the lower and upper molars (respectively, 37% and 36%), followed by the upper and lower premolars (respectively, 18% and 8%).

**Figure 7:**
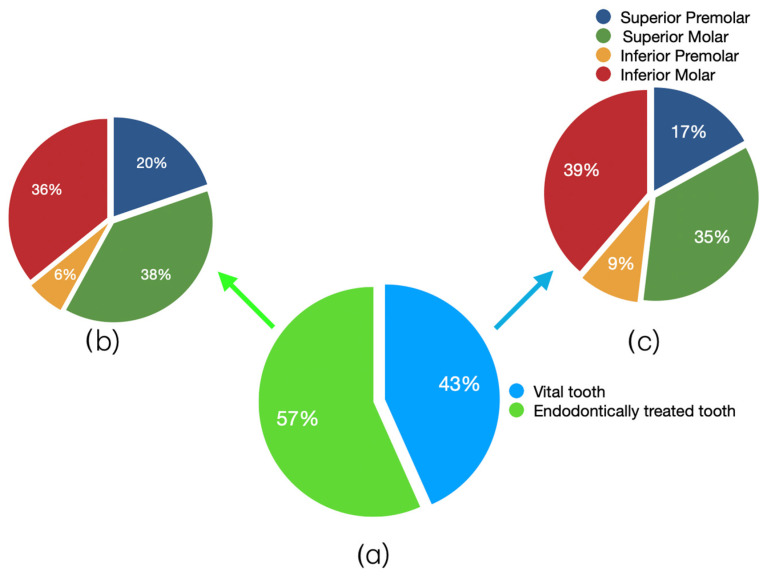
Pie charts. (**a**) Division of the sample into vital and endodontically treated teeth, (**b**) distribution of type of restored teeth in endodontically treated group, (**c**) distribution of type of restored teeth in vital group.

A total of 81 out of the 187 elements (43.32%) were vital at the time of cementation, while 106 (56.68%) were endodontically treated. Figure 7 shows the distribution of the type of restored teeth, divided between vital and endodontically treated teeth. A homogeneous frequency can be observed in the two groups.

### 2.2. Statistical Evaluation

Data analysis was performed using the commercial package IBM SPSS Statistics for Windows (version 29, IBM Corp., Armonk, NY, USA) and the open-source statistical system Jamovi v.2.7.6 (The jamovi project (2025). jamovi. (Version 2.7) [Computer Software]. Retrieved from https://www.jamovi.org, accessed on 2 October 2025). Measures of central tendency, dispersion and shape were calculated for all the variables. Summaries included arithmetic mean, median, standard deviation, interquartile range, minimum, maximum, asymmetry, kurtosis, and the relevant standard errors and 95% confidence intervals. Normality of the data was tested by the Shapiro–Wilk test.

Comparisons between categorical variables in contingency tables were performed using the chi-square test and Fisher’s exact test. The results were considered statistically significant for a *p*-value less than 5% (*p* < 0.05).

Time-to-event analysis was performed considering any occurrence of postoperative complications (sensitivity, secondary decay, crack, decementation, or need for root canal therapy) as an event. Survival distributions between the sandblasting and Er:YAG laser groups were compared using Kaplan–Meier methodology.

## 3. Results

The groups (Er:YAG laser and sandblaster) were comparable with respect to sex distribution and tooth type (*p* = 0.872 and *p* = 0.91, respectively), with standardized mean differences (SMDs) indicating negligible imbalance across these variables (Table 3). Conversely, a significant difference in age was observed between groups, with patients treated using the sandblasting technique being older than those treated with the Er:YAG laser (58.8 ± 12.9 vs. 52.6 ± 14.3 years, *p* = 0.0023; SMD = 0.454).

The mean follow-up was 30 months, with a minimum of 1 year and a maximum of 69 months.

During this period, the recurrence of complications including post-cementation hypersensitivity, debonding, crack, and secondary caries. The incidence of complications was compared between the group in order to establish whether there could be a correlation between the onset of complications and the sandblasting technique.

Table 4 shows the overall frequency of complications. Specifically, in the entire group of 187 adhesively cemented indirect restorations, 3.7% hypersensitivity, 1.07% secondary caries, 0.53% debonding, 0.53% cracking, and the need for root canal therapy at distance were observed in 2.6% of the total.

Comparing the frequency of occurrence in the two groups, the variable that showed the greatest difference was hypersensitivity.

**Figure 8:**
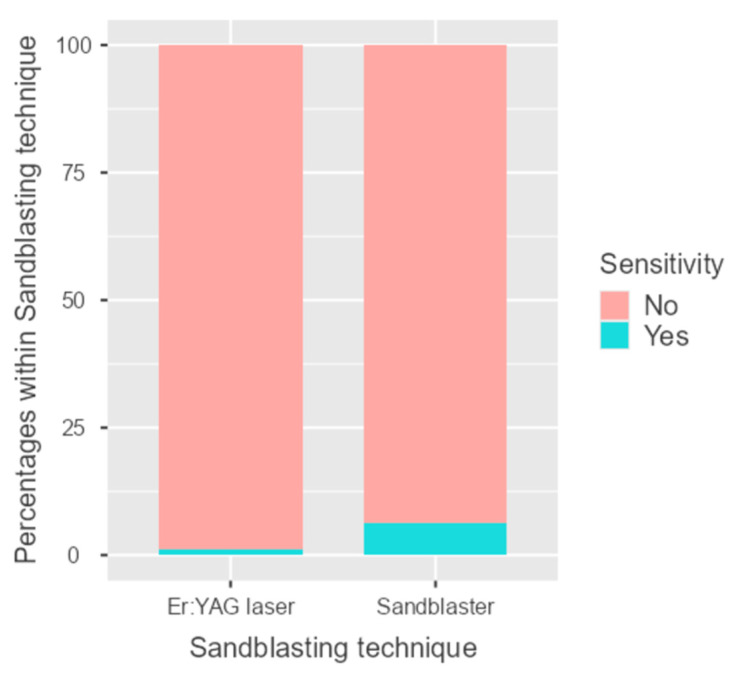
Column chart representing the post-treatment sensitivity event in the two groups (sandblaster and Er:YAG laser).

Specifically, it was present in six cases of the sandblasting group (6.25%) and in only one case of the laser group (1.1%). The difference was borderline significant (*p* = 0.06) (Figure 8, Table 5 and Table 6). In all cases, the increased sensitivity resolved spontaneously 3 months after cementation.

**Figure 9:**
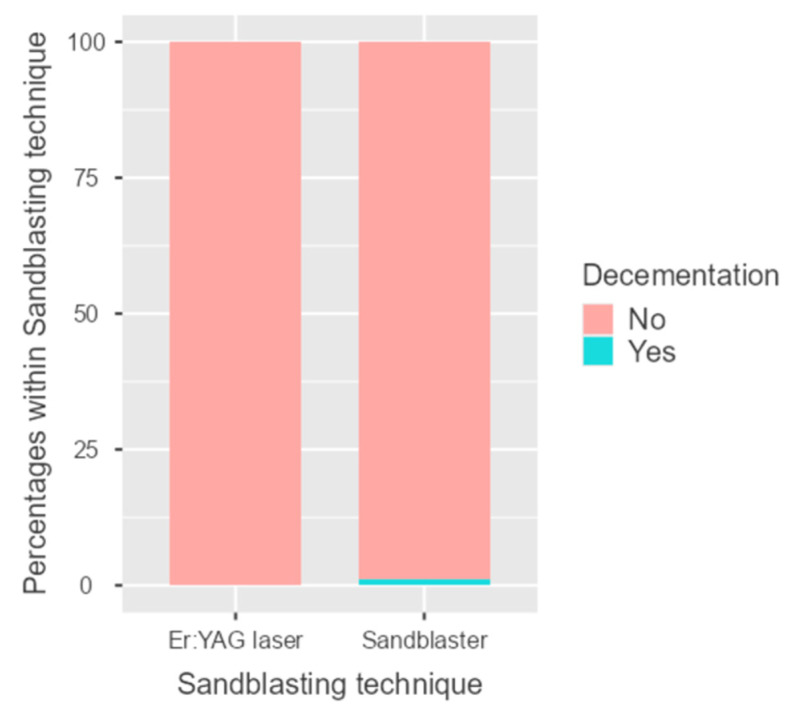
Column graph representing the decementation event in the two groups (sandblaster and Er:YAG laser).

Regarding decementation, only one case was observed in the sandblasting group, and none in the laser group (Figure 9, Table 6 and Table 7). Also, for cracking, there was a single case in the sandblasting group. The difference was not statistically significant (*p* = 0.329).

Secondary decay was detected in two cases, one in the sandblasting group, one in the laser group.

**Figure 10:**
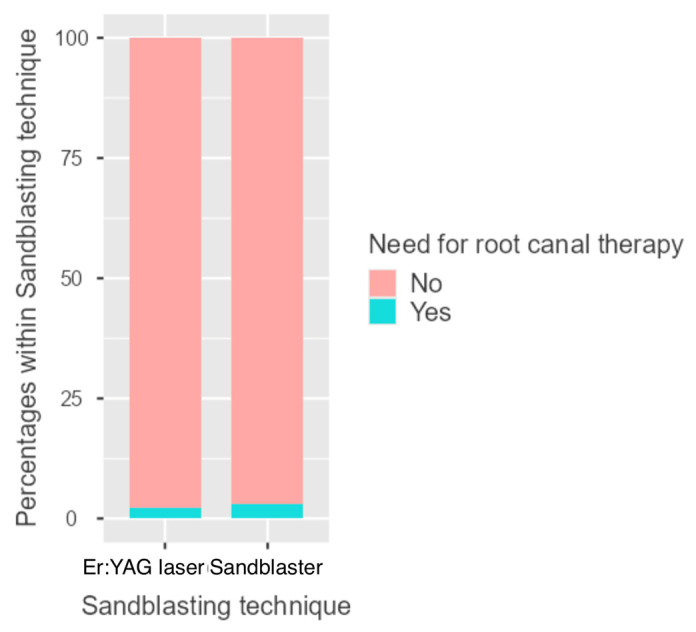
Column chart representing the need of remote root canal treatment event in the two groups (sandblaster and Er:YAG laser).

As regards the need for remote root canal therapy, this occurred in two cases in the laser group (2.2%) and in three cases in the sandblaster group (3.3%) with no statistically significant differences (*p* = 0.694) (Figure 10, Table 6 and Table 8).

All the results described above were assessed in accordance with the FDI classification and are summarized in Table 9. All these properties were evaluated by the same clinician and scored as excellent, good, satisfactory, unsatisfactory (in need of repair) or poor (in need of replacement).

Although the incidence of complications was lower in the Er:YAG group (4.4%) compared to the sandblasting group (12.5%), Kaplan–Meier survival analysis did not show a statistically significant difference between the two techniques (Log-rank test χ^2^ = 2.4864/*p* = 0.1148). The Kaplan–Meier curves of the two conditioning techniques showed comparable survival over time.

**Figure 11:**
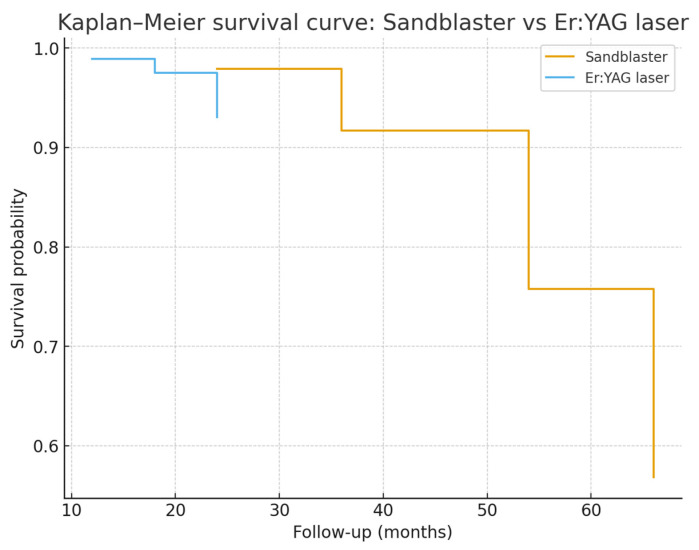
Kaplan–Meier survival curves comparing the cumulative survival of restorations in the sandblasting and Er:YAG laser groups. Survival time is expressed in months. Although the laser group showed fewer failures overall, the log-rank test did not reveal a statistically significant difference between the two curves (x^2^ = 2.49, *p* = 0.115), indicating comparable survival performance throughout the follow-up period.

These results suggest that sandblasting and Er:YAG laser conditioning achieve similar long-term outcomes in terms of restoration durability (Figure 11).

## 4. Discussion

The first consideration that emerges from this large series of adhesively cemented indirect restorations is that the frequency of adverse secondary events is extremely low if an adequate preparation and cementation protocol is applied. The time to time analysis of the two techniques showed a comparable survival over time with a very low failure incidence.

Indirect adhesive restorations fail primarily due to debonding, followed by restoration fractures, secondary caries and, finally, tooth fractures [19,20].

In our series of 187 indirect adhesive restorations, the frequency of secondary caries was 1%, followed by 0.5% debonding and 0.5% cracking of the restoration. No tooth fracture was observed. However, success rates are related to various factors, such as the appropriateness of the adhesive technique, the patient’s caries sensitivity and bad habits, the operator’s experience, and the quality of the materials used.

In this study, bias was reduced by the fact that the same operator performed the preparation and cementation. The same technician performed the restoration, and the same restorative material was used. The steps of the adhesive sequence were always the same, except for the sandblasting/conditioning technique of the build-up. This, in fact, is the variable whose potential effect on the clinical follow-up of the restorations was investigated.

**Figure 12:**
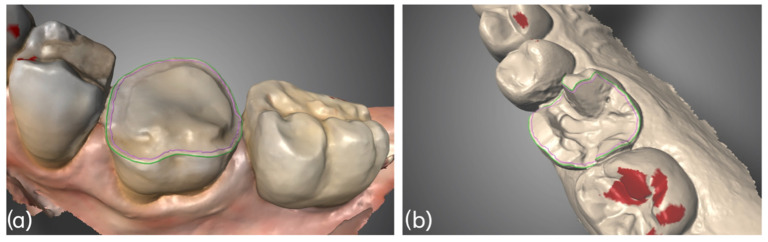
Comparison of build-up preparation techniques for indirect restorations cementation. (**a**) MDPT, (**b**) traditional preparation.

All build-ups were prepared following the principles of the new Morphology Driven Preparation Technique (MDPT). The MDPT aims to preserve as much healthy tissue as possible and minimize exposed dentin. Therefore, the primary bonding layers are the build-up resin and the enamel. Overall, the preparation follows the anatomical shape of the residual tooth and allows for uniform thicknesses, improving both aesthetics and facilitating cementation of the restoration [3]. The butt-join interproximal preparation and the inclined plane (chamfer) on the buccal and palatal/lingual surfaces, compared to traditional preparations, make cementation much more efficient, without creating thicknesses or gaps (Figure 12). This affects the reduction in secondary effects such as cracking, debonding and microleakages.

The adhesive cementation procedure consists of steps for restoration and steps for the build-up. The restoration steps depend on the material. In our series, the material was a CAD/CAM nano-filled ceramic composite. Therefore, the steps were always the same: sandblasting the internal surface of the restoration in the laboratory, application of 37% orthophosphoric acid, rinsing and bonding.

Moreover, steps for the build-up are carried out under a rubber dam and the intraoral sandblasting process results are essential before applying the etching and adhesive. In the literature, it has been widely demonstrated that intra-oral sandblasting significantly improves the efficacy of bond strength.

A recent systematic review and meta-analysis in 2025 demonstrated that air abrasion with 50 µm aluminum oxide particles may improve the bond strength of resin cements to sound dentin, supporting its use before cementing indirect restorations [2].

This technique creates microporosities on the surface, increasing the surface area and improving the wetting of adhesive systems. Compared to no cleaning, air abrasion significantly increased bond strength, supporting its use as an efficient cleaning technique.

Additionally, it outperformed pumice paste, further reinforcing its effectiveness in optimizing adhesive outcomes [2,8].

The same positive effect has been demonstrated on enamel. Specifically, sandblasting with alumina particles has been shown to create a greater roughness on the enamel than dissolving it with conventional acid etching. However, the combination of sandblasting and acid etching has shown a stronger treatment effect on the bonding surface thus produced [21,22]. Regarding choice of sandblasting parameters, Santos et al., in their review, indicated a particle size range from 27 to 50 μm, pressure range from 2 to 3.5 bar, distance from 2 to 10 mm and duration from 5 to 20 s [2].

As with the choice of laser parameters, the gentlest of the effective sandblasting parameters were used. Furthermore, Kui et al., in their study, stated that 27 μm particles produced better results than 50 μm particles [23].

However, despite the demonstrated benefits of aluminum oxide air abrasion on enamel and dentin, Santos et al., in their systematic review, highlighted the challenges associated with its intra-oral application [2]. In vitro studies are typically conducted under ideal conditions, often under fume hoods. These conditions do not reflect the limitations of the oral environment in a clinical setting. Intraoral sandblasting presents risks such as aerosol dispersion, contamination and patient discomfort if not properly isolated [2,24].

In a recent study, Elalfy et al. showed that the use of the Er:YAG laser improves the retention of resin cement on the fiber post surface, while traditional sandblasting reduces the retention of the fiber post on the resin cement [25].

A new build-up conditioning technique has recently been proposed that does not use powders and can therefore potentially overcome the limitations of alumina particle blasting. This technique is based on the use of an Er:YAG laser in QSP mode at very low power. It has been demonstrated that this procedure produces a roughness equal to, or superior to, traditional sandblasting, in the absence of powder and smear layers.

The authors recently published an in vitro study that analyzed both the roughness and morphological analysis of surfaces using ESEM. Surface morphology analysis revealed optimal substrates for adhesion [13]. Specifically, in the laser-treated samples, the build-up composite appeared rougher without a smear layer; the enamel presented clear, well-organized prisms with a honeycomb appearance, also before appliance of acid etching. The dentin appeared free of a smear layer and powder particles, with open tubules and a “fishbone appearance”. The surfaces of the control sample (not sandblasted, not laser-treated) resulted extremely flat, and those treated with a traditional sandblaster were rougher than the control but with a noticeable smear layer covering the tubules and prisms [13].

**Figure 13:**
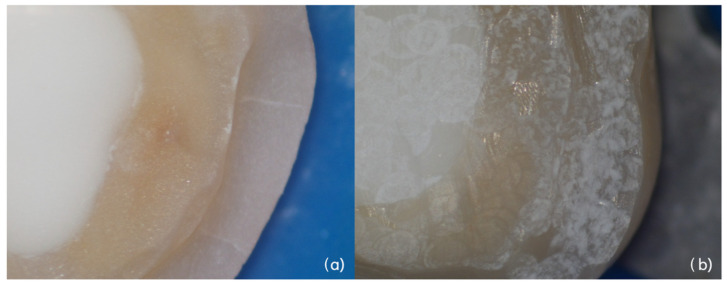
High magnification detail of build-up surface treated with traditional sandblaster (**a**) and Er:YAG laser. (**b**) Increase in roughness is evident. In sample (**b**) there is absence of dust and even in the presence of the laser, the preparation line is maintained.

The ablative action of the Er:YAG laser produces roughness extremely gently, without causing an increase in temperature and without any scattering effect. By analyzing the surfaces obtained using an operating microscope, an increase in roughness can be observed both in those obtained with the sandblaster and laser, but after laser treatment it is evident the absence of dust particle residues and a perfectly preserved preparation line (Figure 13).

The in vitro results obtained need to be clinically confirmed in vivo. Hence, we retrospectively analyzed a very large series, with an average follow-up of 2 years, in order to determine whether the two “sandblasting” techniques resulted in different success rates.

What emerges from our analysis is that, in both cases, the failure rate is very low. The de-bonding and cracking rate was zero among the build-ups treated with the laser and 0.5% among the sandblasted group; secondary leakage was 1% in both groups, with no statistically significant differences.

This confirms the clinical efficacy of sandblasting with alumina powder, with very high long-term success rates if the adhesive protocol is adequate. However, the same success rates can be achieved using laser conditioning, with the advantage of being powder-free. This means greater comfort for the operator and patient, and optimal substrates for a good adhesion.

Post-cementation hypersensitivity requires a separate discussion. Post-cementation hypersensitivity is transient and always resolved within 3 months, so it is not considered a failure of treatment. However, it can impact the patient’s quality of life and clinical success.

It has been widely described in the literature that the type of adhesive used in luting the restoration (etch and rinse or self-etch) does not influence the risk and intensity of post-operative sensitivity [26].

In this case series, a total hypersensitivity rate of 3.7% was observed, in line with the percentages reported in the literature. However, if the frequency is evaluated based on the conditioning technique used, the rate in the sandblasting group was 6% versus only 1% in the laser group.

At present, it is difficult to provide a definitive explanation for this discrepancy. However, there are some bases in the literature that could justify this result. It has been demonstrated from many years that the Er:YAG laser, when applied with a sub-ablative effect of 80 mJ/pulse at 3 Hz, improves discomfort of dentin hypersensitivity immediately and remains at the same level even after a period of 6 months [27,28].

Furthermore, laser-treated enamel has been described to show laser-induced blockage of the organic matrix in the microdiffusion pathway, consistent with the “organic matrix blockage theory.” This translates into a lower recurrence of microleakage and sensitivity [29].

In another study, in an artificial caries model, a significant reduction in secondary caries formation was demonstrated, with a significant 56% reduction in the depth of the primary enamel surface lesion and a 39% reduction in the depth of the root surface lesion, compared to traditional bur and acid-etching techniques [30].

In addition to the well-known anti-hypersensitizing effect, the QSP mode also offers an extremely gentle action compared to traditional Er:YAG laser VSP protocols.

Attrill et al., in their study, established that the Er:YAG laser used with water spray does not produce any damage to the dental pulp within 135 mJ of energy, with a tolerance of up to 160 mJ. The present protocol uses 100 mJ, which is already extremely safe, but using the QSP mode the benefit is greater [14].

Specifically, the single pulse is divided into five pulses. This means that the 100 mJ used acts on the tooth as if 20 mJ were repeated five times. This avoids complications such as thermal overload, scattering, and microcracking on the tooth surface. The Er:YAG laser in QSP mode produces a gentle but efficient effect, eliminating smear layers and powder particles.

## 5. Conclusions

Adhesive techniques and new materials have revolutionized dental rehabilitation, making adhesively cemented indirect posterior restorations a widespread practice.

The success rates of these restorations are very high if the preparation protocol is properly performed, and if all adhesive cementation steps are followed. In particular, it is essential build-up conditioning before etching. Traditionally, this step is performed with an intraoral sandblaster using alumina powder. However, an extremely innovative technique using an Er:YAG laser in QSP mode achieves the same efficacy without smear layer and powder. Furthermore, laser conditioning appears to be associated with reduced post-cement hypersensitivity.

These data need to be confirmed through prospective, randomized, controlled clinical trials. Despite its large cohort and consistently standardized technique, this study remains a retrospective design. It will serve as the basis for a future RCT in which assignment to the laser or sandblasting group will be randomized and outcomes will be assessed with consistent and homogeneous follow-up.

## Figures and Tables

**Table 1:** FDI criteria for the evaluation of dental restoration.

Property		Criteria
Fracture of material and retention	1	No fracture/cracks
	2	Small hairline crack
	3	Two or more or larger hairline cracks and/or material chip fracture non affecting the marginal integrity or approximal contact
	4	Material chip fractures which damage marginal quality or approximal contacts/bulk fracture with partial loss (less than half of the restoration)
	5	Partial or complete loss of restoration or multiple fractures
Marginal adaptation	1	Harmonious outline, no gaps, no white or discolored lines
	2	Marginal gap (<150 µm), white lines/small marginal fracture removable by polishing/slight ditching, slight step/flashes, minor irregularities
	3	Gap < 250 µm not removable/several small marginal fractures/major irregularities, ditching or flash, steps
	4	Gap > 250 µm or dentin/base exposed/Severe ditching or marginal fractures/larger irregularities or steps (repair necessary)
	5	Restoration (complete or partial) is loose but on situ/generalized major gaps or irregularities
Radiographic examination	1	No pathology, harmonious transition between restoration and tooth
	2	Acceptable material excess present/positive/negative step present at margin < 150 µm
	3	Marginal gap < 250 µm/negative steps visible < 250 µm. No adverse effects noticed/poor radiopacity of filling material
	4	Marginal gap > 250 µm/Material excess accessible but not removable/negative step > 250 µm and reparable
	5	Secondary caries, large gaps, large overhangs/apical pathology/fracture/loss of restoration or tooth
Patient’s View	1	Entirely satisfied with aesthetics and function
	2	Satisfied aesthetics/function, e.g., minor roughness
	3	Minor criticism but no adverse clinical effects/aesthetic shortcomings/some lack of chewing comfort/unpleasant treatment procedure
	4	Desire for improvement in aesthetics/function, e.g., tongue irritation; reshaping of anatomic form or refurbishing is possible
	5	Complete dissatisfied and/or adverse effects, including pain
Postoperative (hyper-) sensitivity and tooth vitality	1	No hypersensitivity, normal vitality
	2	Minor hypersensitivity for a limited period of time, normal vitality
	3	Moderate hypersensitivity/delayed/mild sensitivity; No subjective complaints, no treatment needed
	4	Intense hypersensitivity/delayed with minor subjective symptoms/no clinical detectable sensitivity. Intervention necessary, but not replacement
	5	Intense, acute pulpits or non-vital tooth. Endodontic treatment is necessary and restoration has to be replaced
Recurrence of caries (CAR), erosion, abfraction	1	No secondary or primary caries
	2	Small and localized (1) demineralization (2) erosion or (3) abfraction
	3	Larger areas of (1) demineralization (2) erosion or (3) abrasion/abfraction; only preventive measures necessary
	4	Caries with cavitation and suspected undermining caries/erosion in dentin/abfraction in dentin. Localized and accessible, can be repaired
	5	Deep caries or exposed dentin that is not accessible for repair of restoration
Tooth integrity (enamel cracks, tooth fractures)	1	Complete integrity
	2	Smal marginal enamel fracture (<150 µm)/hairline crack in enamel (<150 µm)
	3	Marginal enamel defect < 250 µm/crack < 250 µm; Enamel chipping/multiple cracks
	4	Major marginal enamel defects; gap > 250 µm or dentin or base exposed/large cracks > 250 µm, probe penetrates/large enamel chipping or wall fracture
	5	Cusp or tooth fracture

**Table 2:** Frequency with which each element was treated with indirect restoration within the sample.

Frequencies of Element			
Element	Counts	% of Total	Cumulative %
**14**	1	0.53%	0.53%
**15**	8	4.28%	4.81%
**16**	25	13.37%	18.18%
**17**	10	5.35%	23.53%
**24**	10	5.35%	28.88%
**25**	15	8.02%	36.90%
**26**	25	13.37%	50.27%
**27**	8	4.28%	54.55%
**35**	6	3.21%	57.75%
**36**	18	9.63%	67.38%
**37**	14	7.49%	74.87%
**44**	3	1.60%	76.47%
**45**	6	3.21%	79.68%
**46**	20	10.70%	90.37%
**47**	18	9.63%	100.00%

**Table 3:** Baseline demographic characteristics of patients treated with sandblaster or Er:YAG laser. Potential factors affecting differences between groups were assessed through *p*-values and standardized mean differences (SMD).

Variable	Sandblaster (*n* = 96)	Er:YAG laser (*n* = 91)	*p*-value	SMD
Age, years (mean ± SD)	58.8 ± 12.9	52.6 ± 14.3	0.0023	454
Sex, *n* (%)			872	−45
• Female	57 (59.4%)	52 (57.1%)		
• Male	39 (40.6%)	39 (42.9%)		
Tooth type, n (%)			0.91	
• Upper molar	39 (40.6%)	29 (31.9%)		−100
• Upper premolar	19 (19.8%)	15 (16.5%)		24
• Lower molar	21 (21.9%)	17 (18.7%)		79
• Lower premolar	5 (5.2%)	4 (4.4%)		38

**Table 4:** Frequency of complications divided in sensitivity, decementation, crack, secondary decay, and need for root canal treatment.

Frequencies of Sensitivity				
	**Sensitivity**	**Counts**	**% of Total**	**Cumulative %**
	No	180	96.26%	96.26%
	Yes	7	3.74%	100%
**Frequencies of** **Decementation**				
	**Decementation**	**Counts**	**% of Total**	**Cumulative %**
	No	186	99.47%	99.47%
	Yes	1	0.53%	100%
**Frequencies of Crack**				
	**Crack**	**Counts**	**% of Total**	**Cumulative %**
	No	186	99.47%	99.47%
	Yes	1	0.53%	100%
**Frequencies of Secondary decay**				
	**Secondary decay**	**Counts**	**% of Total**	**Cumulative %**
	No	185	98.93%	98.93%
	Yes	2	1.07%	100%
**Frequencies of Need for root canal therapy**				
	**Need for root canal therapy**	**Counts**	**% of Total**	**Cumulative %**
	No	182	97.33%	97.33%
	Yes	5	2.67%	100%

**Table 5:** Statistical analysis of the variable post-treatment hypersensitivity. Contingency test.

Contingency Tables				
			**Sensitivity**	
**Sanblasting technique**		**No**	**Yes**	**Total**
Er:YAG laser	Observed % within row	90 98.90%	1 1.10%	91 100.00%
Sandblaster	Observed % within row	90 93.75%	6 6.25%	96 100.00%
Total	Observed % within row	180 96.26%	7 3.74%	187 100.00%

**Table 6:** Statistical analysis X^2^ test of the variable sensitivity, decementation and need of root canal treatment.

Sensitivity				
		Value	df	*p*
	X^2^	3.440	1	0.064
	Fisher’s exact test			0.119
	*N*	187		
Decementation				
		Value	df	*p*
	X^2^	0.9530	1	0.392
	Fisher’s exact test			1.000
	*N*	187		
Need for root canal therapy				
		Value	df	*p*
	X^2^	0.1543	1	0.694
	Fisher’s exact test			1.000
	*N*	187		

**Table 7:** Statistical analysis of the variable decementation. Contingency test.

Contingency Tables				
			**Decementation**	
**Sanblasting technique**		**No**	**Yes**	**Total**
Er:YAG laser	Observed % within row	91 100.00%	0 0.00%	91 100.00%
Sandblaster	Observed % within row	95 98.96%	1 1.04%	96 100.00%
Total	Observed % within row	186 99.47%	1 0.53%	187 100.00%

**Table 8:** Statistical analysis of the variable remote need of root canal treatment. Contingency test.

Contingency Tables				
			**Need for root canal therapy**	
**Sanblasting technique**		**No**	**Yes**	**Total**
Er:YAG laser	Observed % within row	89 97.80%	2 2.20%	91 100.00%
Sandblaster	Observed % within row	93 96.86%	3 3.13%	96 100.00%
Total	Observed % within row	182 97.33%	5 2.67%	187 100.00%

**Table 9:** Results assessed in accordance with FDI criteria.

Technique	Sanblaster				Er:YAG laser			
**FDI criteria**	Baseline	3 months	6 months	12 months	Baseline	3 months	6 months	12 months
Decementation								
1	96 (100%)	96 (100%)	96 (100%)	95 (98.96%)	91 (100%)	91 (100%)	91 (100%)	91 (100%)
2	0	0	0	0	0	0	0	0
3	0	0	0	0	0	0	0	0
4	0	0	0	0	0	0	0	0
5	0	0	0	1 (1.04%)	0	0	0	0
Need for root canal therapy								
1	96 (100%)	96 (100%)	96 (100%)	93 (96.88%)	91 (100%)	91 (100%)	91 (100%)	89 (97.80%)
2	0	0	0	0	0	0	0	0
3	0	0	0	0	0	0	0	0
4	0	0	0	3 (3.13%)	0	0	0	2 (2.20%)
5	0	0	0	0	0	0	0	0
Postoperative sensitivity								
1	96 (100%)	90 (93.75%)	96 (100%)	96 (100%)	91 (100%)	90 (98.90%)	91 (100%)	91 (100%)
2	0	6 (6.25%)	0	0	0	1 (1.10%)	0	0
3	0	0	0	0	0	0	0	0
4	0	0	0	0	0	0	0	0
5	0	0	0	0	0	0	0	0
Recurrent caries (Secondary decay)								
1	96 (100%)	96 (100%)	96 (100%)	95 (98.96%)	91 (100%)	91 (100%)	91 (100%)	90 (98.90%)
2	0	0	0	0	0	0	0	0
3	0	0	0	0	0	0	0	0
4	0	0	0	1 (1.04%)	0	0	0	1 (1.10%)
5	0	0	0	0	0	0	0	0
Tooth integrity (Crack)								
1	96 (100%)	96 (100%)	96 (100%)	95 (98.96%)	91 (100%)	91 (100%)	91 (100%)	91 (100%)
2	0	0	0	0	0	0	0	0
3	0	0	0	0	0	0	0	0
4	0	0	0	0	0	0	0	0
5	0	0	0	1 (1.04%)	0	0	0	0

## Data Availability

The original contributions presented in this study are included in the article. Further inquiries can be directed to the corresponding author.

## Abbreviations

The following abbreviations are used in this manuscript:

IDS	Immediate Dentin Sealing
CMR	Cervical Margin Repositioning
MDPT	Morphology Driven Preparation Technique
Er:YAG	Erbium-doped: Yttrium–aluminum–garnet)
QSP	Quantum Square Pulse
